# Bacterial Cellulose-Based Nanocomposites for Wound Healing Applications

**DOI:** 10.3390/polym17091225

**Published:** 2025-04-29

**Authors:** Alexandra-Ionela Dogaru, Ovidiu-Cristian Oprea, Gabriela-Olimpia Isopencu, Adela Banciu, Sorin-Ion Jinga, Cristina Busuioc

**Affiliations:** 1 Faculty of Medical Engineering, National University of Science and Technology POLITEHNICA Bucharest, RO-060042 Bucharest, Romania; alexandra.dogaru01@stud.fim.upb.ro (A.-I.D.); adela.banciu@upb.ro (A.B.); sorin.jinga@upb.com (S.-I.J.); 2 Faculty of Chemical Engineering and Biotechnologies, National University of Science and Technology POLITEHNICA Bucharest, RO-060042 Bucharest, Romania; ovidiu.oprea@upb.ro (O.-C.O.); gabriela.isopencu@upb.ro (G.-O.I.)

**Keywords:** bacterial cellulose, cerium dioxide, *green* synthesis, antibacterial agent, wound healing

## Abstract

Bacterial cellulose (BC) is a polysaccharide produced by Gram-positive and Gram-negative bacteria with a strictly aerobic metabolism, having a huge number of significant applications in the biomedical field. This study investigates the development of bacterial cellulose (BC)-based composite systems that incorporate cerium dioxide nanoparticles (CeO_2_ NPs) used as antibacterial agents to enhance wound healing, particularly for burn treatments. The innovation of this study resides in the integration of CeO_2_ NPs synthesized by using a precipitation method using both chemical and *green* reducing agents, ammonium hydroxide (NH_4_OH) and turmeric extract (TE), in BC membranes composed of ultrathin nanofibers interwoven into a three-dimensional network appearing as a hydrogel mass. Characterization by scanning electron microscopy (SEM), energy dispersive X-ray spectroscopy (EDX), and Fourier-transform infrared spectroscopy (FTIR) confirmed the effective deposition of this agent onto the BC matrix. Antibacterial activity tests against *E. coli* and *B. subtilis* indicated strong inhibition for the composites synthesized following these routes, particularly for the BC-CeO_2_-TE-OH sample, processed by employing both precipitating agents. Cytotoxicity evaluations showed no inhibition of cell activity. Additionally, loading the composites with dexamethasone endowed them with analgesic release over 4 h, as observed through ultraviolet–visible spectroscopy (UV-Vis), while the FTIR spectra revealed a sustained drug presence post-release. These findings highlight BC-based films as promising candidates for advanced wound care and tissue engineering applications.

## 1. Introduction

The skin acts as a protective barrier that screens the body from environmental exposure and ensures a series of vital functions, including thermoregulation, hydration, excretion, and vitamin D synthesis. Damage to the skin can cause a break in epithelial integrity and disrupt the physiological functioning of the underlying tissue, which can lead to immune and inflammatory responses, metabolic changes, and distributive shock, ultimately resulting in multiple organ failure [1]. Moreover, burns are prone to septic complications generated by the colonization of various types of microorganisms that can lead to the formation of a biofilm resistant even to antibiotics administered at concentrations much higher than standard doses [2].

To accelerate the healing process, polymeric dressings have been developed to act as skin substitutes, protect against infections, and support the restoration of skin function [3,4]. These dressings must provide protection against contamination (bacterial or otherwise) and physical damage while allowing for gas exchange, moisture retention, and offering comfort to enhance functional recovery. Additionally, they must control bleeding, absorb wound exudate, accelerate the healing process, and minimize the risk of scar tissue formation [3,4]. While traditional dressings made from materials like cotton or gauze have the disadvantage of drying out the affected area by intensely absorbing wound exudate, recent research has focused on the development of polymer-based materials, in various forms, which provide optimal healing conditions [5].

The treatment process commonly utilizes polymers such as chitin, alginate, collagen, and other biocompatible materials. These biopolymers are highly versatile for tissue engineering, as they degrade into low-toxicity by-products generally well tolerated by living organisms. However, controlling their degradation rates remains a challenge [6]. Among these materials is bacterial cellulose (BC), a polysaccharide produced by various microorganisms, such as bacteria and unicellular fungi with a strictly aerobic metabolism, consisting of glucose molecules linked by β-1,4-glycosidic bonds [7,8]. In other words, BC is a natural linear polymer characterized by a fibrillar morphology consisting of a three-dimensional reticulated network formed by the ribbon-like arrangement of cellulose nanofibrils. Compared to plant-derived cellulose, it exhibits superior properties that have proven to be beneficial in the biomedical field, including its native purity, biocompatibility, and non-toxicity. Despite the fact that the molecular formula of BC is similar to that of plant cellulose, its exceptional physical and mechanical properties arise from its unique three-dimensional structure, which differs significantly from that of the vegetal source, as BC aggregates to form long fibrils with a width of approximately 1.5 nm, thus providing a higher specific surface area, enhanced elasticity, strength, and flexibility. Moreover, BC is chemically equivalent to plant cellulose, but it does not contain secondary products such as lignin, pectin, hemicellulose, or other constituents of lignocellulosic materials [9,10]. The reason for studying this material lies in its ability to provide an ideal three-dimensional substrate for cell attachment, a high-water retention capacity, and the facilitation of gas exchange. Furthermore, BC exhibits tissue healing and regenerative properties, making it an ideal biomaterial for applications in tissue engineering and regenerative medicine [8,11]. Additionally, BC displays excellent water absorption and retention properties, allowing for effective exudate management, while its high mechanical strength in a hydrated state provides structural protection to wounds [8,12,13]. Previous studies have demonstrated that BC acts as a physical barrier, providing protection to the wound against external factors that could delay the healing process. Likewise, due to the abundance of cellulose in nature, the ease of material processing, and the ability to synthesize BC films in various sizes, the development of these dressings is also economically advantageous [14]. BC shows promising potential in various biomedical applications, including the development of wound dressings, artificial skin, controlled drug delivery systems, tissue engineering, and biosensor development [10]. An example demonstrating the applicability of BC in wound healing is the study conducted by Zeng et al. [15]. They synthesized BC membranes functionalized with silver nanoparticles (Ag NPs) through in situ bacterial metabolism by cultivating BC in a culture medium containing the potassium salt D-saccharic acid (SABC). The resulting films (BC-SABC) were then immersed in an Ag NP suspension. The results showed that Ag^+^ ions were rapidly released within the first 72 h, a release rate sufficient to inhibit bacterial growth and thereby accelerate the healing process. Despite the excellent properties demonstrated by BC for wound healing and tissue regeneration, its main limitation is the lack of antimicrobial properties. To overcome this limitation, BC-based composite biomaterials are developed using antimicrobial agents [1,8].

In this study, cerium oxide nanoparticles (CeO_2_ NPs) were selected because CeO_2_, with its fluorite-type, cubic face-centred (FCC) structure, where cerium atoms are located at the centre of the tetrahedron and oxygen atoms at the corners, has been shown to promote wound healing by reducing inflammation, oxidative stress, and infection risk [16,17]. Moreover, Panda et al. reported that CeO_2_ NPs not only protect tissue from various forms of reactive oxygen species (ROS) but also play a role in promoting wound healing [18]. The study conducted by Zhang et al. [16] explored the antibacterial mechanisms of action of CeO_2_ on microorganisms. CeO_2_ NPs directly interact with bacterial membranes through the adsorption of nanostructures on the membrane surface. This adsorption is driven by the electrostatic attraction between the nanoparticles and the negatively charged surfaces of the membranes (at pH around 4). The result of this interaction is the formation of a uniformly distributed CeO_2_ coating around the bacterial membranes. This first stage is followed by a second phase, consisting of oxidative stress induction through reactive oxygen species (ROS), caused by the reversible conversion between Ce^3+^ and Ce^4+^ ions, leading to the chemical degradation of a wide range of organic constituents in microorganisms. Furthermore, it has been demonstrated that the interference between the ions released by the nanoparticles and the nutrient transport proteins in the bacterial cell membrane reduces membrane permeability due to the interaction with the thiol groups (SH^-^) of these proteins. Additionally, the interaction between CeO_2_ NPs and the bacterial membrane may lead to the nanoparticles binding to the mesosome, disrupting vital processes in bacterial proliferation, such as cellular respiration, DNA replication, cell division, and membrane surface growth.

Gofman et al. [19] developed BC-based nanocomposite films with CeO_2_ NPs to evaluate their potential for promoting stem cell proliferation through experiments with murine mesenchymal stem cells. In this study, composite materials with various CeO_2_ concentrations, up to 5%, were prepared and compared with a pure BC substrate. The findings concluded that CeO_2_ NPs positively influence cell attachment and growth on the substrate. Petrova et al. [20] synthesized composite films based on chitosan (CS) and bacterial cellulose (BC) nanofibers doped with CeO_2_ NPs by incorporating disintegrated BC nanofibers and CeO_2_ NPs into the CS matrix. The resulting composites showed potential for use as a support for the growth and proliferation of mesenchymal stem cells, as well as for developing wound dressings for skin lesions. Moreover, the applicability of BC in tissue engineering has been explored in the scientific literature, showing promising results [21,22,23].

The aim of this study was to develop BC-based composite films that include an additional phase of an antibacterial agent. By integrating CeO_2_ NPs as the antibacterial agent, the current study sought to significantly enhance the antibacterial and healing properties of the matrix, with direct applicability in burn treatment. CeO_2_ NPs were in situ synthesized via a precipitation method using cerium ammonium nitrate as a precursor, solubilized in distilled water. To examine the impact of the synthesis parameters, two precipitating agents—ammonium hydroxide and turmeric extract—were employed in this study. This approach enabled a comparison between a chemical synthesis method and a *green* one [24,25]. The motivation in choosing turmeric extract as a *green* reducing agent in the synthesis of CeO_2_ NPs is supported by previous research demonstrating its ability to facilitate nanoparticle formation in an eco-friendly manner. In the study conducted by Patra et al. [26], several advantages of using turmeric extract over conventional chemical methods were highlighted, including low production costs, high bioavailability, the absence of toxicity, and reduced environmental impact. Compared to other *green* reducing agents, turmeric extract contains polyphenolic compounds, particularly curcumin, with strong antioxidant and reducing properties. These compounds promote the formation of cerium oxide nanoparticles by acting as both a precipitating and capping agent [27].

## 2. Materials and Methods

### 2.1. Materials

For the production of BC membranes, the bacterium *Gluconacetobacter saccharivorans* was used. The synthesis process also involved the use of a modified Hestrin–Schramm (MHS) culture medium containing 2% fructose to promote fermentation; for bacterial cell inactivation, an aqueous solution of sodium hydroxide (NaOH, 0.5 N) was employed, as well as distilled water for washing. For the impregnation of BC membranes with the antibacterial agent, the following reagents were employed: cerium ammonium nitrate ((NH_4_)_2_[Ce(NO_3_)_6_], ≥98%, Sigma-Aldrich, St. Louis, MO, USA, *M* = 548.22 g/mol), commercial turmeric powder (Bio Wagner), ethanol (C_2_H_6_O, 96%, Honeywell), and ammonium hydroxide (NH_4_OH, 25%, Sigma-Aldrich, St. Louis, MO, USA).

### 2.2. Synthesis of BC Membranes

The production of BC membranes employed the bacterium *Gluconacetobacter saccharivorans*, which was isolated from apple cider vinegar. To prepare the inoculum, the *Gluconacetobacter saccharivorans* strain was cultivated at a temperature of 30 °C, using a rotary shaker, for a duration of 3 days on a Hestrin–Schramm (MHS) culture medium containing 2% fructose. Subsequently, this inoculum was transferred to a fermentation medium in a 250 mL Erlenmeyer flask, at a ratio of 1:10. Fermentation was carried out in the cultivation medium mentioned above. After 7 days at 27 °C, the films formed in static cultures were removed from the fermentation medium. The obtained films were then treated with a 0.5 N NaOH aqueous solution, at 90 °C, to remove bacterial cells from the BC membrane. The films were subsequently washed several times with distilled water until the pH became neutral. In the next step, the BC membranes were cut into pieces measuring 1.5 × 1.5 cm^2^ and used as wet samples. The protocol has been described in detail in our previously published papers [28,29,30].

### 2.3. Synthesis of Green Precipitating Agent

The first step involved adding 250 mL of ethanol into a round-bottom flask. The solvent was subjected to continuous evaporation through heating, using a hot plate, and the resulting vapours were condensed using a condenser tube. The condensed solvent was gradually collected in an extraction cartridge containing 10 g of commercial turmeric powder. When the liquid reached the overflow level, the solution consisting of solvent and solute was transferred to the distillation vessel. The solute accumulated in the liquid, while the solvent evaporated again. Complete extraction, indicated by a colourless solution, was achieved in approximately 3 h. Turmeric extract is hereafter referred to as TE.

### 2.4. Composite Sample Preparation

(NH_4_)_2_[Ce(NO_3_)_6_] was employed as a precursor for the synthesis of CeO_2_. An appropriate amount of the precursor was weighed, and 100 mL of distilled water was added to obtain a solution with a concentration of 0.1 M. Solubilization was performed through magnetic stirring. The BC membranes were immersed in the solution containing Ce^4+^ ions and allowed to impregnate under magnetic stirring at a temperature of 100 °C for 30 min. Samples were obtained by varying the precipitating agents, as detailed in Table 1. The incorporation was achieved by magnetic stirring at 100 °C for 30 min.

Besides a pure BC sample used as a negative control, a BC film loaded with silver nanoparticles (Ag NPs) was obtained and considered a positive control. Ag NPs were synthesized using silver nitrate (AgNO_3_, Riedel-de-Haën, *M* = 169.87 g/mol) as the precursor. An appropriate amount of precursor was weighed, and 100 mL of distilled water was added to obtain a solution with a concentration of 0.1 M. The BC membranes were immersed in the solution containing Ag^+^ ions and allowed to impregnate under magnetic stirring at a temperature of 100 °C for 30 min. Subsequently, another 10 mL of NH_4_OH was added, and the mixture was magnetically stirred for 30 min.

The BC samples obtained in the previous steps were placed in Petri dishes between two layers of parchment paper to prevent sticking. Additionally, the samples that involved the use of NH_4_OH were washed several times with distilled water until a neutral pH was achieved. The parchment paper was periodically changed until the excess water was removed, and finally, the samples were dried in an oven at 100 °C for 24 h. To maintain the flat surface of the samples, drying was performed under compressive stress. The resultant BC-based composite films possess a thickness in the range of a few micrometres. This characteristic is a direct consequence of the synthesis and drying processes employed, which influence the final material properties, such as mechanical strength, flexibility, and fluid absorption capacity. Such biomaterials exhibit favourable properties for use in wound care applications as thin sheets placed on a support made of textile material, with or without intermediate adhesion layer. The medical device itself was not the subject of this study, as there are many factors and parameters to analyse to establish its final size and shape. Once customized and sterilized, the BC-based dressing can be applied directly onto the wound and possibly secured with a medical bandage. Following this step, the wound site should be monitored, and the dressing changed every several days, depending on the wound condition. In this regard, Orlando et al. [31] reported no adverse effect upon indirect or direct contact with keratinocytes even after 6 days. Importantly, the developed dressings do not require special storage conditions. After the drying process, the samples were placed in plastic containers and kept at room temperature until further analysis was performed.

### 2.5. Characterization Methods

#### 2.5.1. Thermal Analysis

Thermal analysis was conducted to examine the behaviour of the composite systems, as well as the BC native monophase system under varying temperature conditions. The thermal analysis and Fourier-transform infrared (FTIR) spectra of the resultant gases were recorded from room temperature to 900 °C, with a heating rate of 10 °C/min, using NETZSCH STA 449 F3 Jupiter equipment (NETZSCH GmbH, Selb, Germany). Aluminium oxide (Al_2_O_3_) was used as a reference sample.

#### 2.5.2. Fourier-Transform Infrared Spectroscopy

Information about the structure and bonding of chemical species in the analysed materials was obtained through Fourier-transform infrared (FTIR) spectroscopy. FTIR spectra of the composite films were collected using a Thermo Scientific Nicolet iS50 FTIR spectrometer (Thermo Fisher Scientific, Waltham, MA, USA) at wavenumbers ranging from 400 to 4000 cm^−1^ with a resolution of 4 cm^−1^. To obtain these spectra, representative surfaces of the entire sample were placed on the diamond crystal of the instrument, and the attenuated total reflectance (ATR) mode was employed.

#### 2.5.3. X-Ray Diffraction

X-ray diffraction (XRD) was performed to determine the phase composition and crystalline structure of the obtained samples. This analysis was conducted on Shimadzu XRD 6000 equipment (Shimadzu Corporation, Kyoto, Japan) in Bragg–Brentano geometry, with Ni-filtered Cu K*α* radiation (*λ* = 1.54 A), over a 2*θ* range of 10–70°, with a scan step size of 0.02°.

#### 2.5.4. Scanning Electron Microscopy

Using scanning electron microscopy (SEM), it was possible to examine the morphology of the samples with an FEI Quanta Inspect F microscope (FEI Company, Hillsboro, OR, USA). For sample preparation, the specimens were placed on a double-sided carbon adhesive tape that was previously attached to a metal support, and were subsequently coated with pure gold using a Quorum EMS 150R ES sputtering system (Quorum Technologies, Lewes, UK) for 60 s. An accelerating voltage of 30 kV, spot size of 3.5, and working distance of 10 mm were applied.

#### 2.5.5. Energy Dispersive X-Ray Spectroscopy

Chemical composition information about the samples was obtained through energy dispersive X-ray (EDX) spectroscopy, using the same FEI Quanta Inspect F microscope equipped with an EDX detector.

#### 2.5.6. Swelling Tests

The BC-based samples were prepared with standardized shapes and dimensions. Particular attention was given to achieving a narrow mass distribution across the samples. Thus, the BC films were cut into squares with a side length of approximately 1.5 cm. The samples were weighed and subsequently immersed in a solvent (distilled water) at room temperature (20 °C) for various time intervals to observe the swelling behaviour. After each immersion period, the samples were carefully removed, excess water was gently eliminated, and the samples were reweighed to monitor changes in mass over time. The process was repeated for up to 24 h.

#### 2.5.7. Antibacterial Tests

To assess the antibacterial properties of the composite membranes, the following microorganisms were used: Gram-negative bacteria *Escherichia coli* (*E. coli*, strain DH5K, sourced from the microorganism bank of the Bioreactors Laboratory, Faculty of Chemical Engineering and Biotechnologies, National University of Science and Technology POLITEHNCA Bucharest) and Gram-positive bacteria *Bacillus subtilis spizizenii Nakamura* (*B. subtilis*, ATCC 6603).

Antibacterial tests were performed using the disk diffusion method. Nutrient agar (Carl Roth GmbH & Co. KG, Karlsruhe, Germany) was used as the culture medium, with a composition of 0.5% peptone, 0.3% grapevine extract/yeast extract, 0.5% NaCl, and 1.5% agar. The entire culture medium was sterilized by autoclaving at 121 °C for 20 min and then poured into Petri dishes. The plates were inoculated with 0.1 mL of bacterial suspension (the optical density at 600 nm of the inoculum was 0.625 for *E. coli* and 0.452 for *B. subtilis*) using the depletion inoculation technique. The plates were left for approximately 1 h in a humidity-controlled oven to allow for uniform impregnation of the bacterial suspension in the culture medium and to avoid excess liquid in the Petri dishes. The samples were then sterilized under UV light (Portable UV lamp ROTH type IV 254/366 nm) at 256 nm for 30 min. They were aseptically placed on the surface of the medium in the Petri dishes. The samples were incubated at 37 °C for 24 h. The result of the antibacterial activity was measured as the inhibition zone (*IZ*, mm), represented by the clear zone differentiating around the samples containing the active substance.

#### 2.5.8. Cell Tests

##### Direct Contact Method

The in vitro cytotoxicity evaluation was conducted according to ISO 10993 standards [32], primarily aimed at determining the biological response of fibroblast cells following interaction with the composite films. In the first stage, a cell suspension was pipetted into the wells of a culture plate and evenly distributed over their surfaces. The cultures were then incubated at 37 °C until they reached confluence. The culture medium was eventually removed from the plates and replaced with fresh culture medium. The test samples were placed on the cell layer in the centre of each well, covering approximately one-tenth of the surface area of the cell layer. The samples were then incubated at 37 °C for 24 h. The results obtained were observable with a Leica DMi8 optical microscope (Leica Microsystems GmbH, Wetzlar, Germany) and the images were captured using a Leica Flexacam C3 camera (Leica Microsystems GmbH, Wetzlar, Germany). The control sample evaluation was performed in the same manner as for the test samples.

##### Extraction Method

Similar to the previously described method, the assessment of cytotoxicity via the extract method was conducted in accordance with ISO 10993 standards. The extraction conditions applied for this analysis were a temperature of 37 °C for a period of 72 h chosen to closely simulate the conditions under which the dressings would be used. The samples were cut into small pieces to comply with the recommended surface-to-volume ratio in the ISO standards of 6 cm^2^/mL. This ensures that the material is adequately covered in the solvent volume. The samples were then incubated in culture medium under conditions of 5% CO_2_, 20% O_2_, and 95% humidity. Following incubation, the extracts were added to the cell culture, and after 24 h, crystal violet staining (for 20 min) was used for qualitative evaluations. The results obtained were observable with the same Leica DMi8 optical microscope.

#### 2.5.9. Drug Release Profile

To determine the drug release kinetics, dexamethasone (Dex) was used as the active substance. The loading of the chosen composite film was accomplished via soaking in a drug solution obtained by dissolving 5 mg of Dex in 2 mL of ethanol for 24 h after which the sample was dried. The sample was immersed in 10 mL of PBS to simulate physiological release conditions. At predetermined time intervals, a specific volume was collected from the PBS solution, and these samples were analysed using a Jasco V-770 UV-Vis spectrometer (Jasco, Tokyo, Japan). The measurements were conducted over a wavelength range of 200–600 nm.

## 3. Results and Discussion

### 3.1. Material Characterization

#### 3.1.1. Thermal Analysis

Thermal analysis offered valuable information about the degradation behaviour of BC films, which is influenced by factors such as molecular weight, crystallinity, fibre orientation, and the incorporation of additional phases. For pure BC, the degradation occurred in multiple stages, as illustrated in Figure S1A. The initial mass loss of ~6.7% up to 220 °C corresponds to the evaporation of residual or absorbed water and BC dehydration, confirmed by the FTIR gas analysis within this temperature range. The second stage, between 220 and 375 °C, saw a weight reduction of ~52.6%, with the maximum decomposition rate at 345 °C. This phase is linked to dehydration, depolymerization, the breakdown of the polymeric backbone, and the formation of carbonyl and carboxyl groups. At this stage, CO_2_ was the primary gas detected, accompanied by minor CO and H_2_O emissions, marking extensive oxidation. In the final stage (~40.4% weight loss), between 375 and 600 °C, the formation of carbon residue coincided with a strong exothermic effect at 515 °C on the DSC curve, attributed to heat release. Thus, thermal analysis demonstrated the high thermal stability of native BC membranes, a characteristic that could be attributed to the high crystallinity, the highly oriented cellulose chains within the fibrils, and the pure cellulose form.

For the BC-CeO_2_-TE film, a mass loss of ~7.4% occurred near 220 °C, aligned with water elimination (Figure S1B). Subsequent decomposition (~54.8% weigh reduction) took place in the range of 220–330 °C, indicating polymer backbone fragmentation and successive oxidation processes. Moreover, the FTIR results highlight that the main component is CO_2_, accompanied by small amounts of CO and H_2_O, all originating from oxidation reactions. Confirming incomplete oxidation, the spectral bands in the range of 2800–3000 cm^−1^ attributed to the symmetric and asymmetric vibrations of C-H bonds in methyl and methylene groups indicate the presence of small quantities of hydrocarbon fragments in the resulting gases. Above 330 °C, a small weight loss of ~7.1% can be distinguished and attributed to the completion of the combustion process of the residual carbon mass, an aspect also validated by the FTIR diagram. Through a comparative analysis with the pure BC sample, it was observed that the presence of the antibacterial agent, CeO_2_, leads to a decrease in the temperature at which oxidative degradation occurs. Thus, the catalytic effect of the *green* synthesized CeO_2_ on the destruction processes in organic compounds is demonstrated.

The BC-CeO_2_-TE-OH composite exhibited accelerated degradation rates, with an initial mass loss of ~4.3% up to 195 °C due to the evaporation of volatiles, as shown in Figure S1C. The primary degradation phase in the range of 195–215 °C accounted for a ~26.7% weight reduction and a pronounced exothermic peak at 205 °C, with significant CO_2_ release, marking the oxidation process. It is also worth mentioning that this process is characterized by an accelerated degradation, an effect confirmed by the sharp appearance of the DTG curve. It was observed that the polymer skeleton was degraded, fragmented, and oxidized at much lower temperatures, this phenomenon being associated with the presence of a higher amount of CeO_2_, ensured by using two precipitating agents. This assertion is consistent with the FTIR analyses performed on the modified BC samples, which confirms the deposition of a considerable amount of antibacterial agent on the BC membrane compared to the other samples analysed (Figure 3). In addition, the SEM and EDX investigations confirmed that the addition of two precipitating agents guaranteed a significant increase in the number of particles formed and attached on the surface of the BC fibrils. The secondary degradation phase took place up to 330 °C, decreasing the weight by ~17.4%, while the final phase lasted until 600 °C, with another ~17.3% mass loss, through a new complex exothermic process, with the maximum achieved at 330 °C. The FTIR results confirm the presence of significant amounts of CO_2_ and a reduced content of CO and H_2_O, but also some hydrocarbon fragments. Compared to the previously investigated samples, in the case of the BC-CeO_2_-TE-OH system, the analysis of the DTG curve could indicate that the degradation process proceeds much faster, as evidenced by the narrowed appearance of the peaks. This effect could be assigned to the distribution of CeO_2_ NPs on the surface of the BC fibrils.

The most peculiar decomposition occurred in the case of the BC-CeO_2_-OH material (Figure S1D). As before, a small mass loss of ~4.6% emerged in the temperature range of 100–170 °C, together with a weak endothermic effect on the DSC curve. Between 170 and 230 °C, the main thermal degradation stage took place, recording a weight reduction of ~37.4%, a process associated with a strong exothermic effect on the DSC curve at 200 °C, an overall lower value compared to the BC-CeO_2_-TE-OH film, for which two such processes were identified. This phenomenon could be explained by the high amount of CeO_2_ NPs deposited on the BC membrane, as confirmed by the FTIR analyses recorded on the modified BC samples (Figure 3). Moreover, the DTG curve indicates that the degradation process occurred instantaneously, a fact confirmed by the sharp appearance of the graph, reaching a maximum rate at 195 °C. Above 230 °C, a decrease of ~12.9% in mass was observed, this process being accompanied by multiple weak exothermic effects. The FTIR diagram of the resulting gases validated the fragmentation of the polymer chains, as well as the start of the oxidation process of these fragments after 190 °C, when significant amounts of CO_2_ and a reduced content of CO and H_2_O were observed.

In the case of the BC-Ag-OH sample, thermal degradation occurred in stages, with an initial mass loss of ~5.4% between 20 and 240 °C, associated with water removal and partial decomposition of Ag_2_O (Figure S1E). The main BC degradation step (~54.5% weight reduction) occurred in the range of 240–390 °C, with a peak at 345 °C, followed by carbon residue combustion between 390 and 600 °C, when a major mass decrease of ~36.4% was recorded and the strongest exothermic peak emerged at 475 °C. It seems that the presence of Ag NPs on the surface of the BC fibril stabilizes, to a certain extent, the composite by maintaining BC membrane integrity at higher temperatures compared to the CeO_2_ phase.

**Figure 1:**
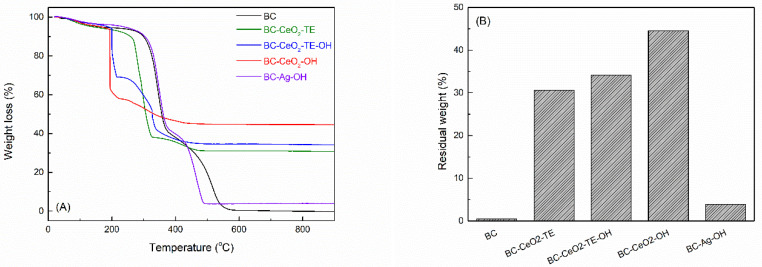
(**A**) Thermo-gravimetric curves and (**B**) graphical representation of residual mass after thermal degradation up to 900 °C for pure BC and composite samples.

Comparing mass losses among samples and according to the graphs displayed in Figure 1, the highest values of the total weight loss were recorded for the BC and BC-Ag-OH samples, approximately ~99.7 and ~96.1%, respectively, confirming the reduced amount of Ag NPs deposited on the surface of the native BC membrane (Table 2). In contrast, for CeO_2_-based nanocomposites, the mass losses were significantly lower. The lowest ones were observed for BC-CeO_2_-OH and BC-CeO_2_-TE-OH, ~55.5 and ~65.8%, respectively. These results are consistent with those presented later and obtained from the antibacterial tests, which demonstrated increased efficiency for the BC-CeO_2_-TE-OH sample (Figure 9). In conclusion, a rough estimation of antibacterial agent loading was made from the thermal analysis curves, and an approximate proportion of CeO_2_ NPs was determined considering that the combustion residue after heating to 900 °C represents the stable oxide phase (Table 2). As expected, the use of ammonium hydroxide as a precipitating agent triggers the deposition of a higher proportion of the antibacterial phase. However, the precipitation efficiency is reasonable even when the *green* precipitating agent is used alone (30.6% residue).

#### 3.1.2. EDX Spectroscopy

**Figure 2:**
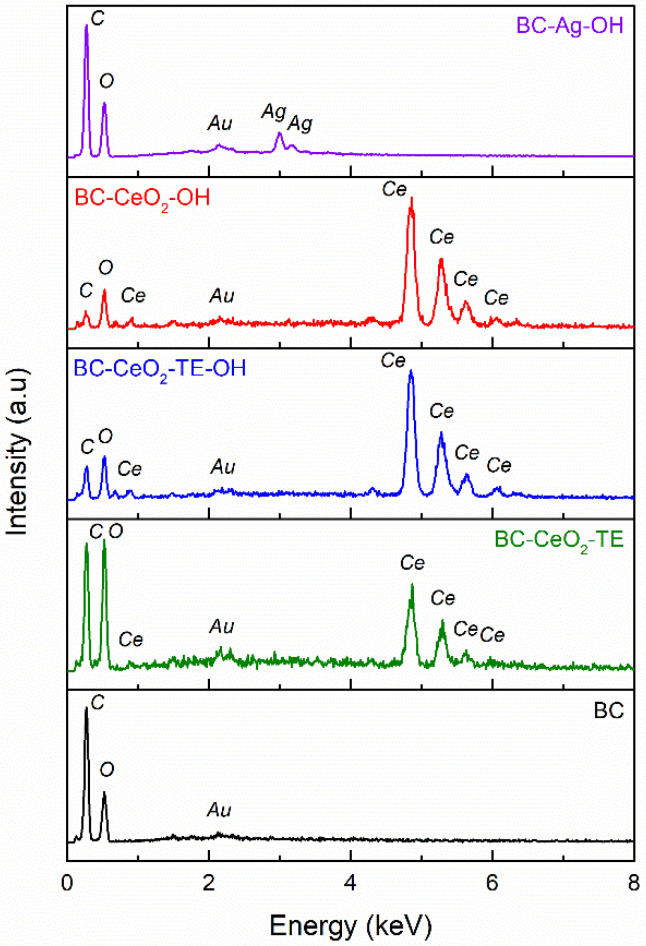
EDX spectra of pure BC and composite samples.

The chemical/elemental composition of the obtained samples can be examined through EDX, with the results of this analysis presented in Figure 2. The data achieved for the pure BC membrane demonstrated the presence of carbon (C) and oxygen (O) atoms in the material structure. Furthermore, the results for the samples containing CeO_2_ confirmed the presence of cerium (Ce) atoms, as well as the absence of other foreign elements in the material composition, leading to the conclusion that the samples produced do not contain impurities. The BC-CeO_2_-TE composite exhibits a higher content of carbon and oxygen compared to the control sample (pure BC), a feature attributed to the additional presence of organic components related to the employed *green* precipitating agent (TE).

The BC-CeO_2_-TE-OH film is characterized by a significantly higher cerium content, along with a decrease in carbon and oxygen concentration. This observation can be correlated with the results provided by the FTIR data, suggesting the deposition of a larger amount of CeO_2_ on the surface of the BC membrane. Similar results were observed for the BC-CeO_2_-OH sample, with the only difference being that the proportion of carbon is diminished since TE was no longer used. In the case of the BC-Ag-OH sample, as expected, the spectrum indicates the presence of carbon, oxygen, and silver (Ag) atoms at the membrane surface.

Fair quantitative information on the antibacterial agent content is difficult to determine, as the loading of the BC support was performed in situ, employing a precipitation method with two reducing agents, instead of directly incorporating previously synthesized CeO_2_ NPs. Given the biogenic nature of BC, the corresponding membranes may have different intrinsic characteristics, which could lead to different loading degrees over large areas, in other words to inhomogeneous deposition. Moreover, not all precipitated CeO_2_ NPs remain attached to the BC matrix, part of the oxide phase settling to the bottom of the reaction beaker. Under these conditions, it is difficult to correctly quantify the concentration of the cerium element. Starting from the EDX spectra recorded on a large zone, quantitative data were extracted and are centralized in Table 3. However, data analysis must take into account that this investigation was performed on those areas with rich loading and cannot be considered representative of the entire sample since the CeO_2_ layer found at the surface can shield the signal coming from the organic component.

#### 3.1.3. FTIR Spectroscopy

**Figure 3:**
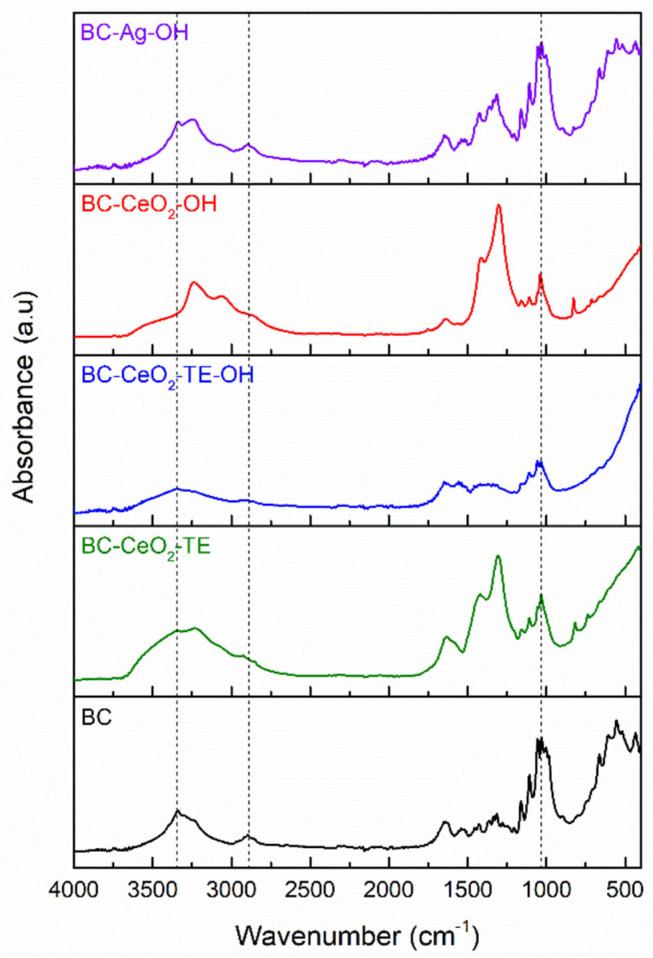
FTIR spectra of pure BC and composite samples.

FTIR spectra were recorded for both pure and modified BC membranes, and the results can be observed in the graphs shown in Figure 3. In the case of pure BC sample, the polymer fingerprint is highlighted by the specific vibrational bands corresponding to O-H bonds, located at approximately 3341 and 1640 cm^−1^, the vibrational band of C-H bonds at approximately 2895 cm^−1^, and the vibrational bands located at 1426, 1361, and 1315 cm^−1^, which are characteristic of -CH_2_, -CH_3_, and O-H groups or bonds, respectively [33]. When CeO_2_ is added to the system, an enhancement in the bands in the range of 3050–2950 cm^−1^ and at 1033 cm^−1^ can be observed. This may indicate the formation of cerium hydroxide (Ce(OH)_4_) or cerium oxyhydroxides due to precipitation, suggesting an increase in the concentration of OH^-^ groups, and, on the another hand, possible interactions between BC and cerium-based compounds [34]. Additionally, the emergence of new vibrational bands at 1304 cm^−1^ and below 750 cm^−1^, corresponding to Ce-O bonds, was also noted [35,36].

In the case of the BC-CeO_2_-TE-OH composite, the appearance of the FTIR spectrum is entirely different from that of the other samples, in that the most intense vibrational band occurs at wavenumbers below 750 cm^−1^, with the contributions generated by pure BC being diminished. This change may be attributed to the deposition of a significant amount of CeO_2_ onto the BC membrane, which screens the bands specific to the substrate [37]. The increased intensity of the vibrational band observed at lower wavenumbers is further confirmed in the spectrum of the BC-CeO_2_-TE sample, followed by the BC-CeO_2_-OH sample, thereby demonstrating the effectiveness of the two precipitating agents. However, it appears that the maximum precipitation efficiency occurred when TE was combined with ammonium hydroxide. In the case of Ag deposition, no major differences in the reference spectrum are observed, indicating that there is no chemical interaction between the Ag NPs and the functional groups in BC. It is likely that the Ag NPs are merely electrostatically attached to the surface of the fibrils or trapped within the membrane volume [38,39].

#### 3.1.4. XRD Analysis

**Figure 4:**
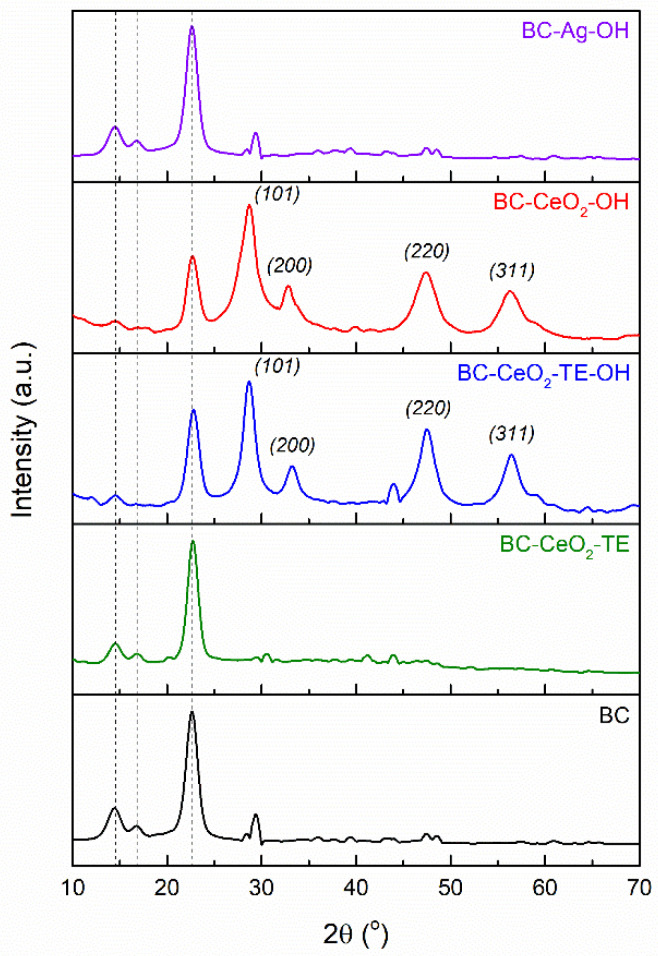
XRD patterns of pure BC and composite samples.

The XRD patterns of the developed composites are presented in Figure 4. In the case of the pure BC sample, three characteristic diffraction peaks were observed at 2*θ* = 14.5, 16.8, and 22.7°, associated with diffraction on the (101), (101-), and (002) planes, respectively, according to the scientific literature [40,41]. Furthermore, the data from the literature attribute these diffraction peaks to a typical cellulose I structure [40,41]. For the BC-CeO_2_-TE film, a significant reduction (3.5 times) in the absolute intensity of the three major diffraction peaks typical of the pure BC sample was identified, resulting from the impregnation of the BC membrane with Ce-based compounds, as well as different organic components from TE. However, the general appearance of the diffractogram in this region remained unchanged. This intensity decrease is likely due to a screening effect caused by the phases deposited during the synthesis process [21]. Since no additional crystalline phases were highlighted when TE was employed as the precipitating agent, cerium is probably integrated in amorphous or low-crystallinity compounds based on Ce-O bonds, as demonstrated by the corresponding FTIR spectrum (Figure 3). Following the use of ammonium hydroxide in the BC-CeO_2_-OH and BC-CeO_2_-TE-OH samples, a decrease (around 15 times) in the absolute intensity of the diffraction peak specific to the BC sample at 22.7° was observed, which can be attributed to a pronounced screening effect caused by a thicker layer of the antibacterial agent. Moreover, supplementary diffraction peaks were observed at 28.6, 33.1, 47.4, and 56.3°, which are characteristic of the cubic structure of CeO_2_ ceramic (ICDD 00-081-0792). This observation is consistent with the subsequent results provided by the antibacterial test (Figure 9). Regarding the BC-Ag-OH material, the diffraction pattern is similar to that of the pure BC membrane, suggesting that the deposition occurred in a relatively small quantity and the corresponding crystalline phase cannot be evidenced by diffractometric means. This hypothesis is supported by previous analyses, such as FTIR spectroscopy (Figure 3).

#### 3.1.5. SEM Analysis

**Figure 5:**
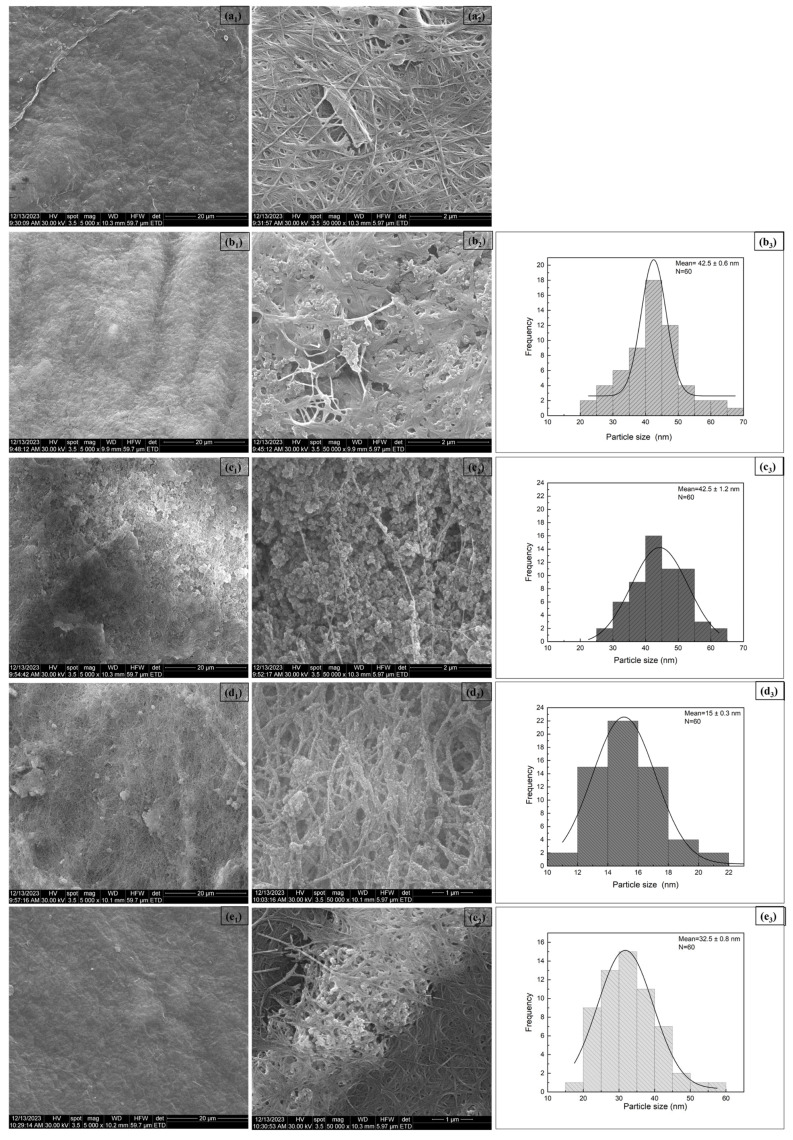
(1 and 2) SEM images and (3) particle size distribution histograms for (a) pure BC, (b) BC-CeO_2_-TE, (c) BC-CeO_2_-TE-OH, (d) BC-CeO_2_-OH, and (e) BC-Ag-OH samples.

The results of the SEM investigation are illustrated in Figure 5. Pure BC consists of randomly arranged nanofibers forming a dense, porous, three-dimensional network (dried under laboratory conditions), as shown in Figure 5(a_2_). To evaluate the efficiency of the synthesis method in relation to particle size, after loading the BC membranes with CeO_2_, size distribution histograms of the particles impregnated on these supports were also considered. These histograms were generated by performing multiple measurements using the JMicroVision application, followed by applying the Gaussian model to obtain the average values. After CeO_2_ NP deposition using TE as the precipitating agent, spherical nanoparticles with a diameter of about 42 nm are clearly visible, embedded within the BC membrane structure, whose loose appearance is preserved (Figure 5(b_2_)). On the other hand, the addition of NH_4_OH during the synthesis of the samples significantly increased the quantity of nanoparticles formed. The CeO_2_ NPs appear as aggregates approximately 42 nm in size, made of noticeably smaller entities than those observed in other experimental conditions, predominantly located between the BC fibrils (Figure 5(c_2_)). Such behaviour can be attributed to the presence of TE, which could play the role of a capping agent through its components, stabilizing the nanoparticle interface and further inhibiting the possible over-growth process of extended aggregation. Moreover, this demonstrates a correlation between the visualised morphology and the antibacterial properties, which were further highlighted by the antibacterial test (Figure 9). As has been demonstrated many times in the scientific literature, the effectiveness against bacteria depends largely on the size of the proposed nanostructures, with a decrease in size leading to an increase in the targeted effect. In the case of the BC-CeO_2_-OH composite, CeO_2_ NPs were found to create an almost continuous coating over the BC fibrils (Figure 5(d_2_)), even though the individual nanoparticles are around 15 nm, leading to weaker antibacterial properties compared to the other samples, as demonstrated in subsequent testing (Figure 9), probably due to pronounced agglomeration. It appears that smaller particles have a stronger effect on Gram-positive bacteria [40,42]. However, no activity was detected against the *B. subtilis* strain, suggesting that processes of nucleation and growth or the implication of the BC support are of capital importance when it comes to surface-related processes, like bacterial inhibition or killing. For the BC-Ag-OH sample, random and uneven attachment of Ag NPs to the BC membrane is seen (Figure 5(e_2_)), forming aggregates of nanoparticles with a size of approximately 32 nm.

These experimental results are supported by previous research studies, where similar findings were reported, such as those in the study by Chen et al. [41]. In their paper, CeO_2_ NPs were obtained through precipitation, using an ammonium hydroxide solution as the precipitating agent. The resulting nanoparticles exhibited a spherical morphology, forming aggregates with sizes ranging from 9.4 to 18.8 nm, depending on pressure and temperature conditions. Similarly, Cui et al. [43] synthesized CeO_2_ NPs using Ce(NO_3_)_3_·6H_2_O as the precursor, showing a decrease in particle size with the increase in cerium source concentration, starting at 58.2 nm. The results for the BC-Ag-OH system are comparable to those obtained by Asif et al. [44], who produced Ag NPs with a spherical morphology and particle size distribution in the range of 10–25 nm.

BC is one of the best candidates for biogenic nanomaterials, being directly produced at the nanoscale by certain bacteria, which makes it inherently nanostructured from the outset. Thus, it is composed of ultrafine cellulose fibres (fibrils as individual threads of crystalline cellulose chains, or ribbons as bundles of fibrils twisted or layered together), typically in the range of 10–100 nm in diameter. These nanofibers form a highly porous three-dimensional network, very suitable as support for the deposition of other nanostructured phases, as was previously demonstrated in the case of CeO_2_ NPs loaded on BC membranes. According to the SEM investigation and derived size distribution histograms (Figure 5), the oxide phase also falls within the nanometric domain (average particle sizes between 15 and 43 nm), which makes both components of the composites nanosized, justifying in this way the term nanocomposites.

### 3.2. Sorption/Desorption Capability

#### 3.2.1. Swelling Tests

**Figure 6:**
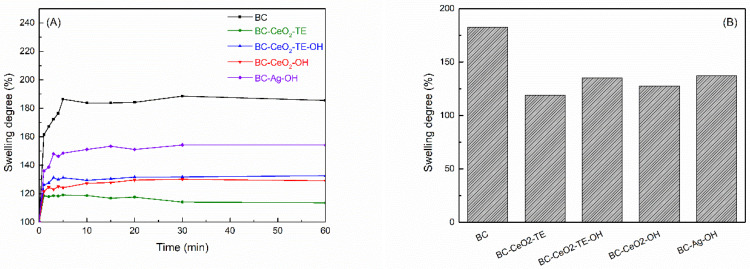
(**A**) Swelling kinetics up to 60 min and (**B**) swelling degree at 24 h for analysed samples.

The data obtained from this investigation are graphically represented in Figure 6. As shown in Figure 6A, the swelling tests revealed a sharp increase in the swelling degree during the first few minutes after the samples were immersed in distilled water, corresponding to rapid liquid adsorption, followed by stabilization of this increase over time. Additionally, it was observed that the highest swelling degree was associated with the pure BC sample, while the swelling levels of the composites were influenced by the presence of additional phases and the synthesis method used. The swelling behaviour of BC films is largely determined by the presence of hydrophilic functional groups such as hydroxyl (OH^−^), which facilitates hydrogen bonding with water molecules. The incorporation of antibacterial agents into the polymeric matrix may lead to the modification of the polymeric structure, thereby influencing the water absorption capacity by disrupting the fibrillar network surface and intermolecular interaction. First, the deposited oxide layer could act as a mechanical shield, preventing the access of water molecules to the BC surface, and second, an electrostatic interactivity could occur between the non-bonding electrons from CeO_2_ and the hydroxyl groups on the surface of the BC fibrils, greatly reducing the available sites for binding to water molecules. Among the modified BC membranes, the BC-Ag-OH sample exhibited the highest swelling degree, while the BC-CeO_2_-TE sample had the lowest. This indicates that precipitation was the most efficient for the CeO_2_ NPs synthesized using the *green* method, whereas Ag NP loading was characterized by the lowest yield. It should also be noted that such behaviour could also be correlated with the attachment of different molecules from TE to the surface of the BC fibrils, blocking water retention by limiting the H-bonding ability. Furthermore, the swelling values after 24 h, as depicted in Figure 6B, are consistent with the previously presented findings.

#### 3.2.2. Drug Release

**Figure 7:**
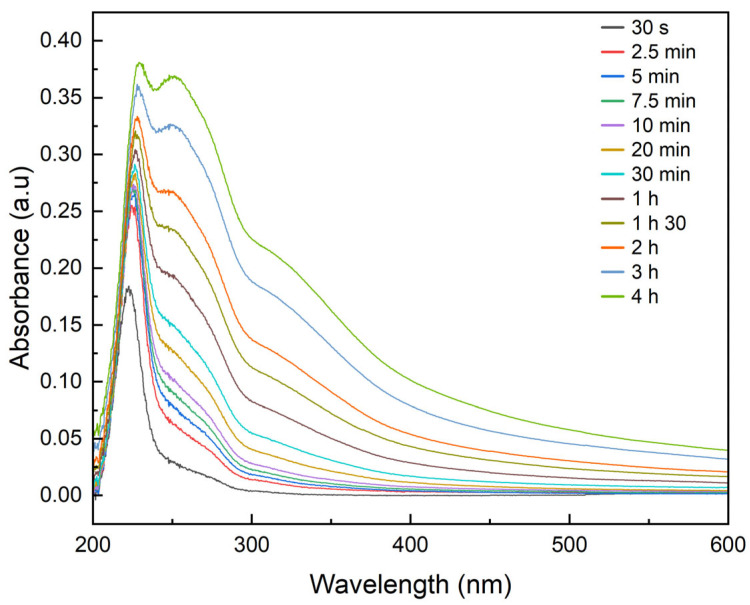
UV-Vis spectra corresponding to the release profile of Dex from the BC-CeO_2_-TE-OH sample.

**Figure 8:**
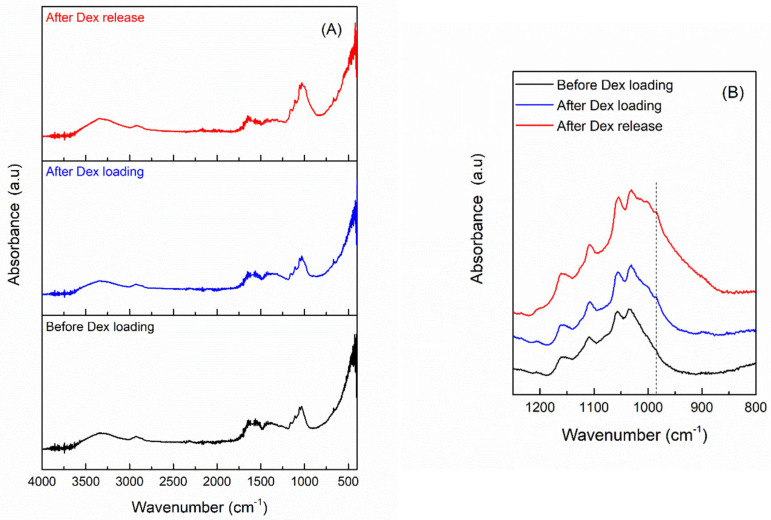
(**A**) FTIR spectra of the BC-CeO_2_-TE-OH sample before and after Dex loading, as well as after a 4 h release period and (**B**) representation over a narrow wavenumber range.

The UV-Vis spectra obtained after immersing the BC-CeO_2_-TE-OH sample loaded with Dex in PBS solution with the aim of evaluating the release kinetics of the active substance are presented in Figure 7. The measurements indicate a gradual release of the active substance over a period of 4 h, suggesting a possible covalent interaction between the polymer matrix and the drug. These results are further supported by the FTIR spectra obtained before and after drug loading, as well as after drug release (Figure 8). In Figure 8B, a magnified view of the region of interest is presented, highlighting detailed spectral changes that confirm the successful loading of Dex in the sample. Thus, the FTIR spectrum of the composite after the drug loading procedure indicates that the presence of Dex through the shoulder emerged at 985 cm^−1^, attributed to the axial deformation of C-H bonds, as reported in the literature [45,46]. Moreover, the FTIR data recorded after a 4 h release period display a similar profile to that obtained after active substance loading, demonstrating that Dex remains present in a significant amount within the sample, further ensuring a slow release of analgesic molecules.

### 3.3. Biological Characterization

#### 3.3.1. Antibacterial Testing

**Figure 9:**
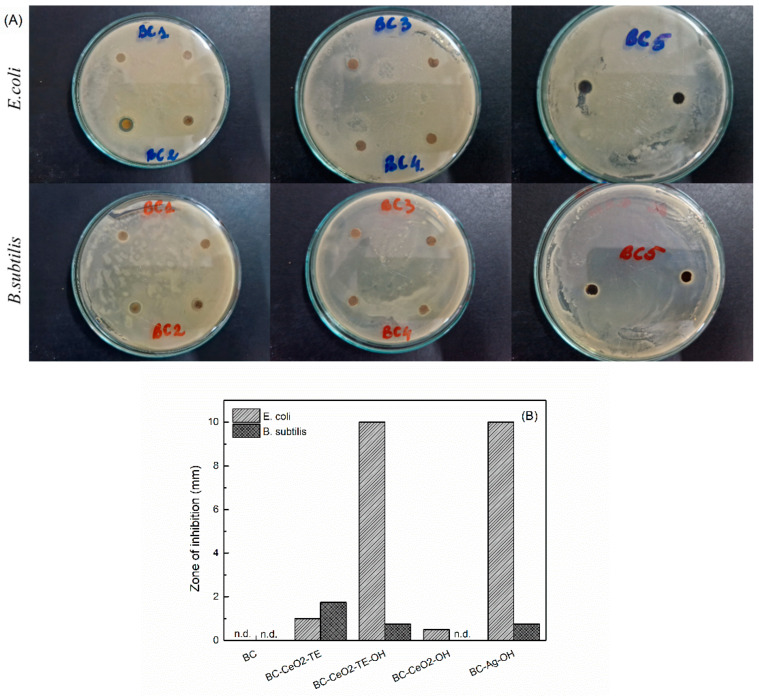
(**A**) Digital images and (**B**) graphical representation showing antibacterial activity of pure BC and composite samples against *E. coli* and *B. subtilis*.

The action of the deposited antibacterial agents can be observed in Figure 9. In the case of the pure BC (BC1), no measurable inhibition zone was detected against the used bacteria, demonstrating that the antibacterial activity of the obtained dressings is exclusively due to the presence of CeO_2_ NPs within the BC matrix. Among all of the samples analysed, BC-CeO_2_-TE-OH (BC3) and BC-Ag-OH (BC5) exhibited the strongest inhibitory effect against the growth of *E. coli*, with inhibition zones exceeding 10 mm. For *B. subtilis*, the inhibition zones were approximately 0.75 mm. In contrast, the BC-CeO_2_-TE (BC2) composite was found to be more effective against *B. subtilis*. The difference in effect is attributed to the increased susceptibility of *E. coli* to the reactive oxygen species (ROS) generated by the antibacterial agent. This is due to the structure of the cell wall, which is easier to penetrate in Gram-negative bacteria than in Gram-positive bacteria. Comparing the results with the BC-Ag-OH (BC5) film, it can be concluded that the BC-CeO_2_-TE-OH (BC3) composite possesses similar properties against Gram-negative bacteria.

Similar results have been obtained in other research approaches investigating the inhibitory effect of CeO_2_ NPs against *E. coli* and *S. aureus*, showing low sensitivity for *S. aureus* (diffusion halos ranging from 0.53 to 3.33 mm) [47]. A comparable trend was also observed for Ag NPs, where the inhibition zone for *E. coli* exceeded 15 mm [48]. Studies conducted to determine the mechanism of action of CeO_2_ NPs on bacteria have demonstrated that, following electrostatic interaction, they are adsorbed onto the bacterial membrane surface, forming a uniform coating. This interaction leads to oxidative stress (ROS), induced by the reversible conversion from Ce^3+^ to Ce^4+^, and reduces cell membrane permeability [16]. For Ag NPs, the exact mechanism of action on bacteria is not fully understood. However, it is hypothesized that, following physical interaction with the cellular surface of various bacteria, the nanoparticles adhere to the cell wall surface and enter cells through porin channels, disrupting intracellular organelles and biomolecules and inducing ROS [49].

Future studies will focus on determining the optimal concentration of the antibacterial agent, ensuring that it effectively targets and disrupts the bacterial cellular structure without adversely affecting the integrity of the tissue cells. Such research will include a broader range of bacterial strains, including both *Gram*-positive and *Gram*-negative bacteria that will provide a more comprehensive understanding of the potential impact that these dressings have in combating infections in burn tissue.

#### 3.3.2. Cellular Protocols

##### Direct Contact Method

**Figure 10:**
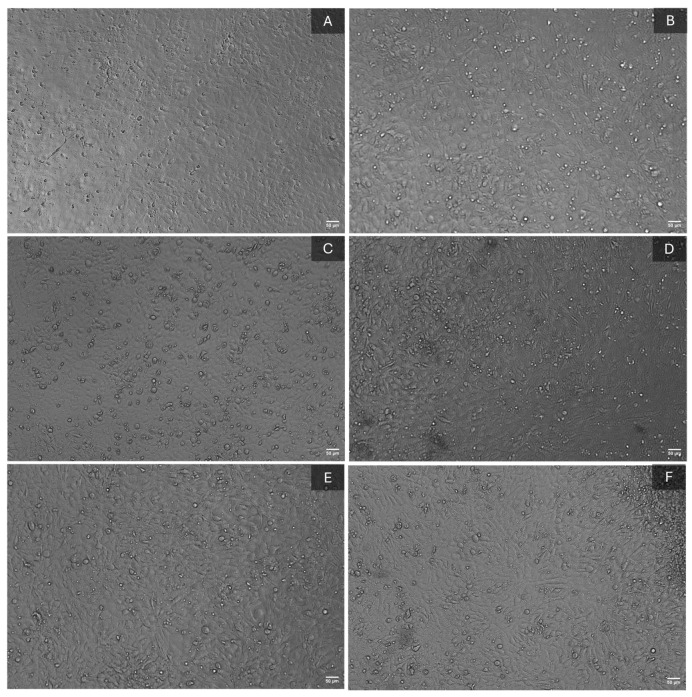
Cytotoxicity evaluation using the direct contact method: (**A**) control, (**B**) pure BC, (**C**) BC-CeO_2_-TE, (**D**) BC-CeO_2_-TE-OH, (**E**) BC-CeO_2_-OH, and (**F**) BC-Ag-OH samples.

The results gathered after applying this protocol are highlighted in Figure 10. As shown in Figure 10B, in the case of the pure BC sample, compared to the control sample (Figure 10A), cells adhered to the surface, as demonstrated by the changes in cell morphology from a spherical to an elongated shape. The formation of actin filaments at the cellular membrane level was observed as well, acting as cellular extensions that facilitate adhesion to the substrate. Furthermore, the adhered cells displayed a multilayered arrangement. Similar characteristics were observed for the BC-CeO_2_-TE composite (Figure 10C). However, the most promising results are highlighted in Figure 10D, where both the previously described morphologies and the presence of a significantly reduced number of non-adhered cells on the surface of the BC-CeO_2_-TE-OH material can be noted, demonstrating the biological properties of the dressing. Moreover, these results align with those obtained from the FTIR investigation, where better loading of the BC membrane with the antibacterial agent was observed (Figure 3). A promising biological response was also obtained for the BC-CeO_2_-OH sample (Figure 10E).

##### Extraction Method

**Figure 11:**
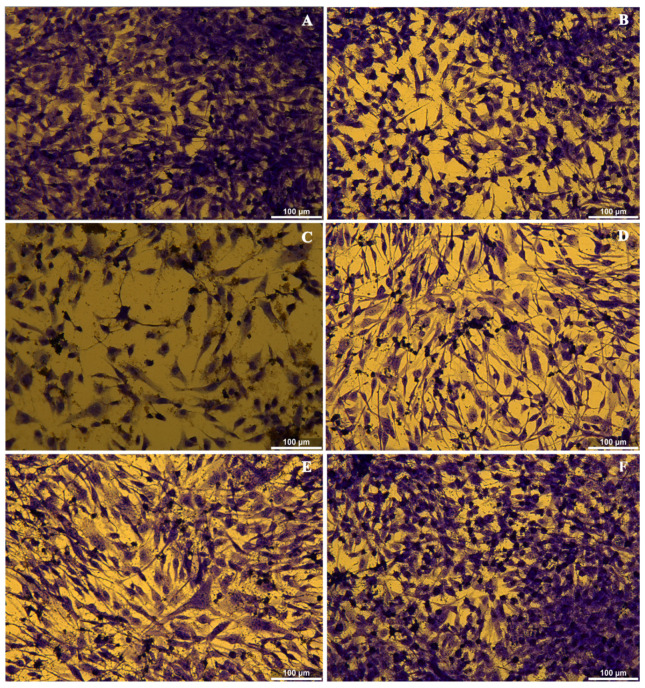
Cytotoxicity evaluation using the extraction method: (**A**) control, (**B**) pure BC, (**C**) BC-CeO_2_-TE, (**D**) BC-CeO_2_-TE-OH, (**E**) BC-CeO_2_-OH, and (**F**) BC-Ag-OH samples.

Figure 11 displays the data of the biological evaluation performed on the obtained composite systems in the presence of fibroblast cells. Microscopy images of the pure BC sample indicate a slight decrease in cell density compared to the control sample. Furthermore, it was observed that the cells did not undergo any morphological change, maintaining their typical elongated shape associated with cellular adhesion to the substrate. The best results were achieved following the addition of the antibacterial agent to the system, in the case of BC-CeO_2_-TE-OH and BC-CeO_2_-OH composites, as no qualitative differences in morphology were observed compared to the control sample, and the cell distribution demonstrates the formation of a homogeneous monolayer. Thus, it was confirmed that the dressings do not inhibit cellular activity.

The shelf life of the potentially developed commercial products based on such nanocomposites could be estimated depending on the size and shape of the medical device, the packaging method, the type of packaging, the preservation atmosphere, etc. Due to such a large number of variables, it is difficult to estimate a shelf life now. At this stage of the study, we can state that the film, in a dry state with minimal water activity in the BC matrix, did not show fungal infestation, even if it was maintained in an open atmosphere, the supply of nanoparticles allowing the water activity in the matrix to be maintained low (there was no variation in the film humidity depending on the humidity of the environment).

The significant role of CeO_2_ NPs in improving wound closure, minimizing scarring, alleviating inflammation, and exerting antibacterial activity has attracted extensive attention from the research field [50]. Moreover, a growing importance has been attributed to *green* synthesis methods for CeO_2_ NPs, as these ensure precise control over surface chemistry, particle size, and overall medical impact. Besides BC, other polymeric matrices have been assessed and composited with Ce-based phases with the aim of revealing new antibacterial materials for wound repair applications. Gobi et al. [51] discussed the effect of incorporating CeO_2_ NPs into chitosan (CS) and polyvinyl alcohol (PVA) films by using the solution casting technique, with a focus on achieving excellent antibacterial action against wound infesting pathogens and no cytotoxicity to fibroblast cells. Elabbasy et al. [52] proposed a film casting route for enhancing traditional bandages by starting from a polymeric matrix based on PVA and hyaluronic acid (HA) and then incorporating hydroxyapatite (HAP) and cerium vanadate (Ce_3_(VO_4_)_4_) NPs to accelerate the healing process and prevent bacterial infections during prolonged recovery periods. The proven features of Ce-containing phases combined with the well-known advantages triggered by the BC membranes (purity and crystallinity, nanostructuring, water-holding capacity, flexibility and resistance, reactivity and degradability, biocompatibility) pave the way for a synergistic approach that could take the healing process to another level.

## 4. Conclusions

In conclusion, the aim of this study was to develop bacterial cellulose-based wound dressings by combining a polymeric matrix with two antibacterial agents, cerium dioxide and silver nanoparticles, for the treatment of skin lesions. The composite films were successfully synthesized by incorporating the antibacterial agent into the bacterial cellulose matrix. An innovative aspect of this work is the combination of bacterial cellulose, cerium dioxide, and turmeric extract, in comparison with other existing scientific studies, as well as the use of two precipitating agents. The samples were characterized using a series of techniques to determine their morphological aspects and to validate the deposition of the antibacterial agents onto the substrate. Among the tested samples, the BC-CeO_2_-TE-OH composite, synthesized using turmeric extract and ammonium hydroxide as precipitating agents, exhibited the most significant antibacterial activity against *E. coli* and *B. subtilis,* while maintaining cellular compatibility, as shown by the cell viability test. Additionally, the incorporation of dexamethasone to provide analgesic properties was successful and the release profile was determined, indicating gradual release of the drug over 4 h.

Considering these findings, it can be affirmed that the composite films developed in this study have promising potential in the field of tissue engineering. Future efforts could focus on modifying the method of impregnating bacterial cellulose membranes with antibacterial agents or on adding an additional phase to enhance efficiency. Additionally, the impregnation of these membranes with another antibacterial agent, such as vanadium pentoxide, presents significant potential, which could open new perspectives for the development of advanced materials for medical applications.

## Figures and Tables

**Table 1:** Samples obtained by using different precipitating agents.

Sample Code	Precursor	Precipitating Agent
BC-CeO_2_-TE	(NH_4_)_2_[Ce(NO_3_)_6_]	50 mL TE
BC-CeO_2_-TE-OH		50 mL TE + 10 mL NH_4_OH
BC-CeO_2_-OH		10 mL NH_4_OH
BC-Ag-OH	AgNO_3_	10 mL NH_4_OH

**Table 2:** Quantitative data extracted from thermal analysis curves.

Sample Code	Organic Component (%)	Residue (%)
BC	99.7	0.3
BC-CeO_2_-TE	69.4	30.6
BC-CeO_2_-TE-OH	65.8	34.2
BC-CeO_2_-OH	55.5	44.5
BC-Ag-OH	96.1	3.9

**Table 3:** Quantitative data extracted from EDX spectra.

Sample Code	C (wt%)	Ce (wt%)	O (wt%)
BC-CeO_2_-TE	7.5	59.4	33.1
BC-CeO_2_-TE-OH	27.6	53.0	19.4
BC-CeO_2_-OH	29.0	47.3	23.7

## Data Availability

The original contributions presented in this study are included in the article/Supplementary Material. Further inquiries can be directed to the corresponding author.

## Supplementary Materials

The following supporting information can be downloaded at: https://www.mdpi.com/article/10.3390/polym17091225/s1, Figure S1. (a) Complex thermal analysis and (b,c) FTIR spectra of evolved gases for: (A) pure BC, (B) BC-CeO_2_-TE, (C) BC-CeO_2_-TE-OH, (D) BC-CeO_2_-OH, and (E) BC-Ag-OH samples.
