# Supplementary material for: Bacterial Cellulose-Based Nanocomposites for Wound Healing Applications
Source: Polymers (Basel). 2025 Apr 29;17(9):1225. doi: 10.3390/polym17091225 (PMC12073685; doi:10.3390/polym17091225)
Supplement: Supplementary file 1 [file polymers-17-01225-s001.zip › polymers-3550918-supplementary.pdf]

Supplementary Material

Bacterial Cellulose-Based Nanocomposites for Wound Healing Applications

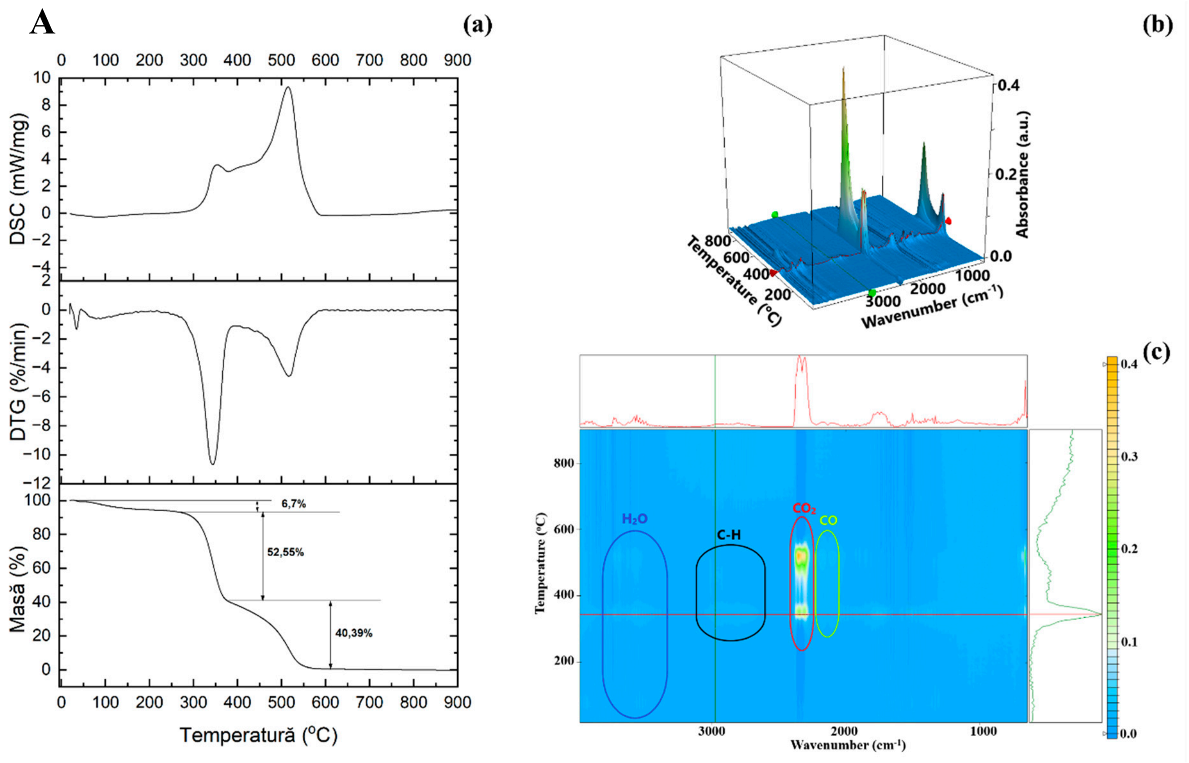

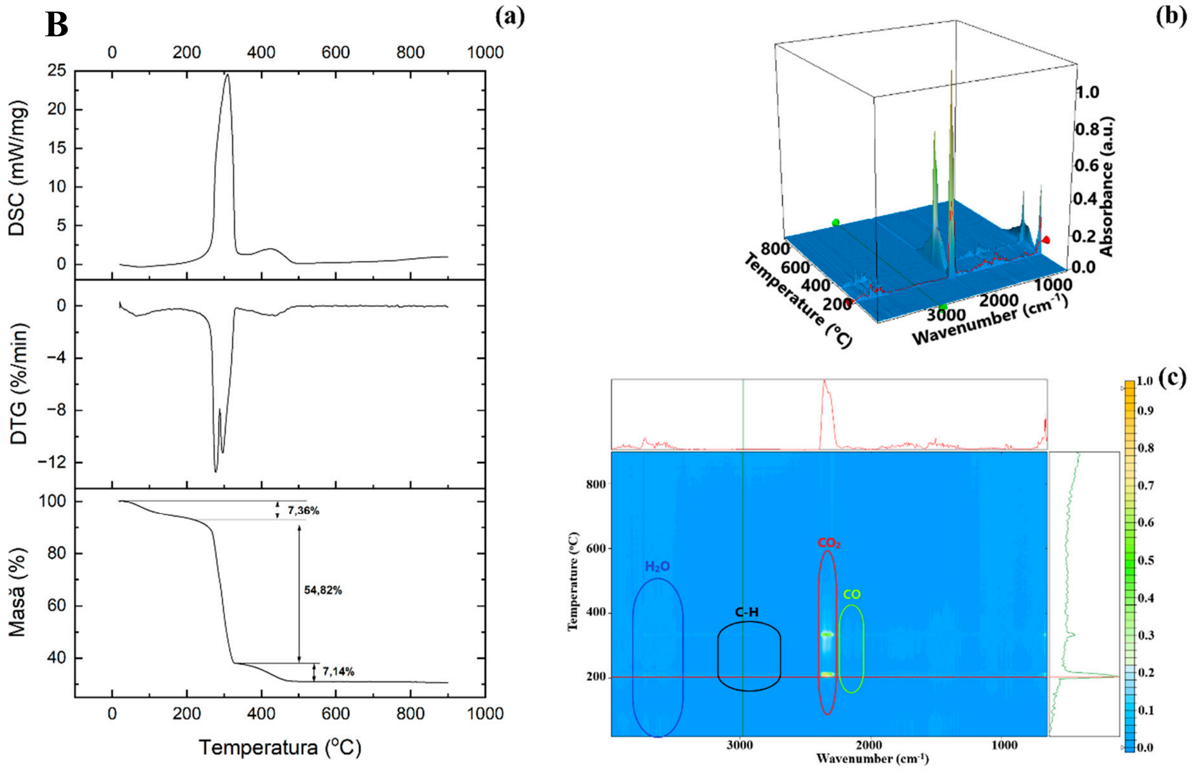

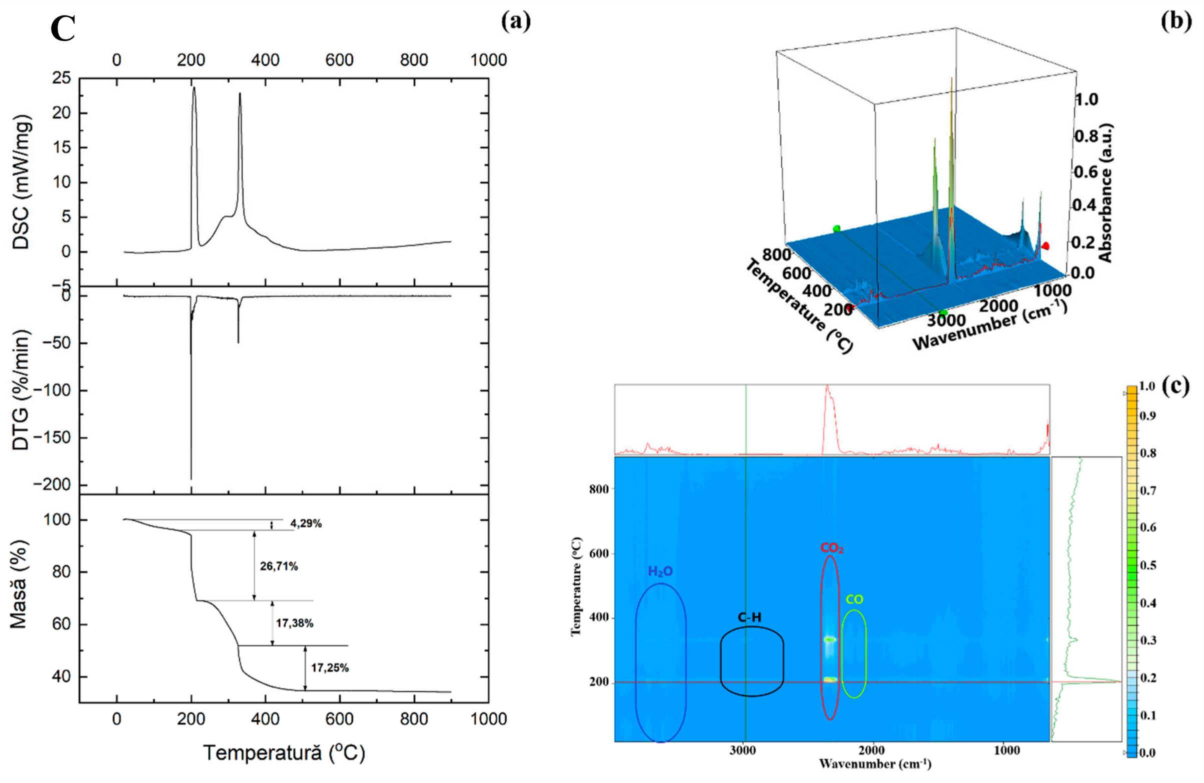
C
(a)
(b)
(c)
DSC (mW/mg)
DTG (%/min)
Masă (%)
Temperatură (°C)
4,29%
26,71%
17,38%
17,25%
Absorbance (a.u.)
Temperature (°C)
Wavenumber (cm⁻¹)
H₂O
C-H
CO₂
CO

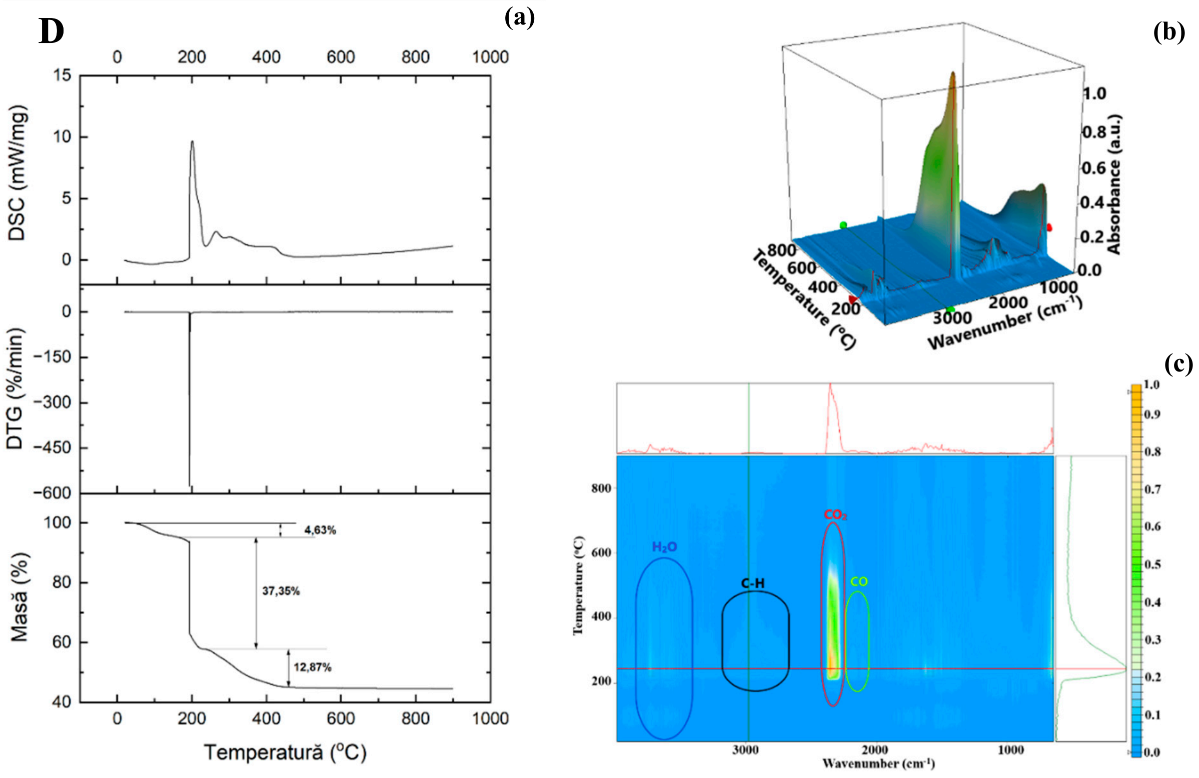
D
(a)
(b)
(c)
DSC (mW/mg)
DTG (%/min)
Masă (%)
Temperatură (°C)
4,63%
37,35%
12,87%
Absorbance (a.u.)
Temperature (°C)
Wavenumber (cm⁻¹)
H₂O
C-H
CO₂
CO

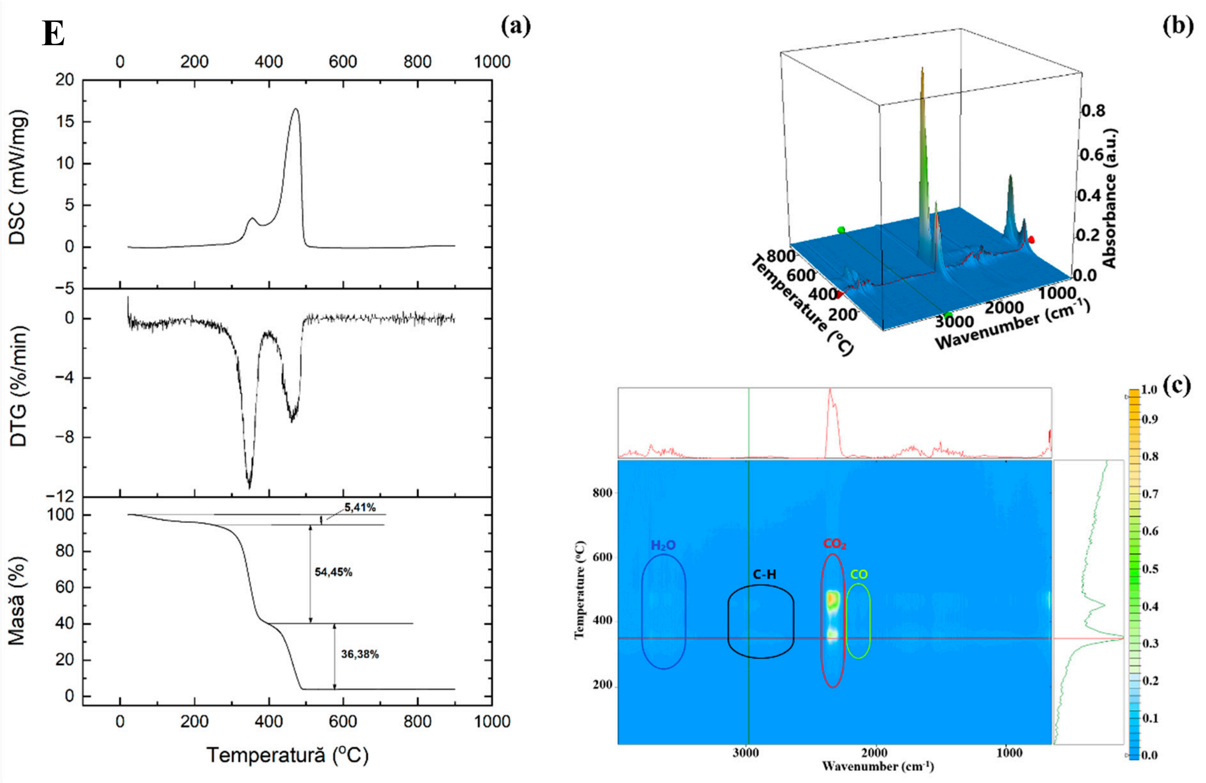

Figure S1. *(a) Complex thermal analysis and (b and c) FTIR spectra of evolved gases for: (A) pure BC, (B) BC-CeO$_2$-TE, (C) BC-CeO$_2$-TE-OH, (D) BC-CeO$_2$-OH, and (E) BC-Ag-OH samples.*
